# Hantavirus inhibits apoptosis by preventing mitochondrial membrane potential loss through up-regulation of the pro-survival factor BCL-2

**DOI:** 10.1371/journal.ppat.1008297

**Published:** 2020-02-07

**Authors:** Carles Solà-Riera, Marina García, Hans-Gustaf Ljunggren, Jonas Klingström

**Affiliations:** Department of Medicine Huddinge, Center for Infectious Medicine, Karolinska Institutet, Karolinska University Hospital, Stockholm, Sweden; Division of Clinical Research, UNITED STATES

## Abstract

Hantaviruses, zoonotic RNA viruses belonging to the order *Bunyavirales*, cause two severe acute diseases in humans, hemorrhagic fever with renal syndrome (HFRS) and hantavirus pulmonary syndrome (HPS). Hantavirus-infected patients show strong cytotoxic lymphocyte responses and hyperinflammation; however, infected cells remain mostly intact. Hantaviruses were recently shown to inhibit apoptosis in infected cells. By inhibiting granzyme B- and TRAIL-mediated apoptosis, hantaviruses specifically and efficiently inhibit cytotoxic lymphocyte-mediated killing of infected cells. Hantaviruses also strongly inhibit apoptosis triggered intrinsically; i.e., initiated through intracellular activation pathways different from those used by cytotoxic lymphocytes. However, insights into the latter mechanisms are currently largely unknown. Here, we dissected the mechanism behind how hantavirus infection, represented by the HFRS-causing Hantaan virus and the HPS-causing Andes virus, results in resistance to staurosporine-induced apoptosis. Less active caspase-8 and caspase-9, and consequently less active caspase-3, was observed in infected compared to uninfected staurosporine-exposed cells. While staurosporine-exposed uninfected cells showed massive release of pro-apoptotic cytochrome C into the cytosol, this was not observed in infected cells. Further, hantaviruses prevented activation of BAX and mitochondrial outer membrane permeabilization (MOMP). In parallel, a significant increase in levels of the pro-survival factor BCL-2 was observed in hantavirus-infected cells. Importantly, direct inhibition of BCL-2 by the inhibitor ABT-737, as well as silencing of BCL-2 by siRNA, resulted in apoptosis in staurosporine-exposed hantavirus-infected cells. Overall, we here provide a tentative mechanism by which hantaviruses protect infected cells from intrinsic apoptosis at the mitochondrial level by inducing an increased expression of the pro-survival factor BCL-2, thereby preventing MOMPs and subsequent activation of caspases. The variety of mechanisms used by hantaviruses to ensure survival of infected cells likely contribute to the persistent infection in natural hosts and may play a role in immunopathogenesis of HFRS and HPS in humans.

## Author summary

Hantaviruses cause two severe, often fatal, diseases in humans: hemorrhagic fever with renal syndrome (HFRS) and hantavirus pulmonary syndrome (HPS; also called hantavirus cardiopulmonary syndrome (HCPS)). Two hallmarks of human hantavirus infection are robust immune cell activation and hyperinflammation. Despite these strong immune responses, hantavirus-infected cells do not succumb to cell death in patients. Recent studies have shown that hantaviruses hamper cytotoxic lymphocyte-mediated killing, by inhibiting cytotoxic granule-dependent induction of apoptosis and TRAIL-mediated apoptosis, as well as inhibiting intrinsic apoptosis. However, mechanisms behind hantavirus induced inhibition of intrinsic apoptosis have not been described. Here, we show that hantavirus infection leads to increased production of the anti-apoptotic protein BCL-2, hampering the permeabilization of mitochondria and thereby blocking downstream signaling and activation of caspases. Treatment of infected cells with a BCL-2 inhibitor, as well as silencing of BCL-2 with siRNA, both reverted the anti-apoptotic effect. Taken together, this study reveals new insights into the interactions between hantaviruses and infected cells and demonstrates novel mechanisms by which hantaviruses inhibit apoptosis by hampering the permeabilization of mitochondria.

## Introduction

Orthohantaviruses (hereafter referred to as hantaviruses) are emerging zoonotic viruses of the order *Bunyavirales* with a worldwide distribution which are carried by a plethora of different natural hosts such as small mammals, reptiles and fish [1, 2]. Hantavirus infection of their natural hosts is asymptomatic, and hosts remain persistently infected and act as long-term virus reservoirs. In contrast, in humans rodent-borne hantaviruses cause hemorrhagic fever with renal syndrome (HFRS) in Eurasia and hantavirus pulmonary syndrome (HPS; also called hantavirus cardiopulmonary syndrome (HCPS)) in the Americas, two lethal diseases for which there currently is no specific treatment nor an FDA-approved vaccine [3–7]. The majority of HFRS cases are associated with Hantaan virus (HTNV), but also other hantaviruses, including Puumala virus, Seoul virus, and Dobrava virus cause HFRS with up to 12% case fatality rates [5, 8]. The severest cases of hantavirus-associated diseases are related to the HPS-causing Andes virus (ANDV) and Sin Nombre virus in South and North America, respectively. These highly pathogenic hantaviruses are associated with case fatality rates of 35–40% [6, 9, 10]. Transmission to humans occurs mainly by inhalation of excreta from infected rodents [4, 5], but human-to-human transmission of ANDV can also occur and cause concerning outbreaks [11, 12].

Apoptosis represents a well-regulated mechanism to eliminate virus-infected cells, thereby restricting the dissemination of a pathogen throughout the body and contributing to clearing the infection. It can be triggered extrinsically, by cytotoxic lymphocytes specifically targeting the infected cells, or intrinsically, by the infected cell *per se* after sensing different cell stress signals. Cytotoxic lymphocytes can kill target cells via cytotoxic granule-dependent induction of apoptosis, relying on the enzyme granzyme B that is mainly expressed by cytotoxic lymphocytes. Another unique capacity of cytotoxic lymphocytes is to activate extrinsic apoptosis through the interaction of specific cytotoxic lymphocyte ligands, such as FasL, TRAIL, and TNF, with specific death receptors (DRs), such as Fas, DR4, DR5 and TNF-R1, on infected target cells [13, 14]. But apoptosis can also be activated within an infected cell. The intrinsic apoptosis pathways, which can be activated by a virus infection, DNA damage, chemotherapeutic agents or serum starvation [15, 16], lead to mitochondrial outer membrane permeabilization (MOMP) causing the release of pro-apoptotic factors such as cytochrome C (cyt C) into the cytosol [17]. Cyt C is then involved in the activation of the caspase cascade, ultimately resulting in activation of the executioner caspase-3 [18]. The B-cell lymphoma 2 (BCL-2) family of proteins control cell fate mainly by direct physical interactions that regulate MOMP [19, 20]. These interactions, and their resulting consequences, depend on the affinity and the relative abundance of the different pro- and anti-apoptotic BCL-2 family members. Upon a particular cell stressor, the composition and activation state of both pro- and anti-apoptotic BCL-2 family proteins change; thus apoptosis-inducing pathways stimulate a pro-apoptotic effect, leading to increased MOMP and the release of cyt C into the cytosol [21–25]. Anti-apoptotic BCL-2 family members, mainly BCL-2 and BCL-X_L_, inhibit MOMP, thereby inhibiting the release of cyt C from mitochondria and the subsequent activation of caspases [26].

Common hallmarks of HFRS and HPS are increased capillary leakage, hyperinflammation, and strong immune cell activation including elevated levels of activated natural killer (NK) cells [27] and virus-specific cytotoxic CD8^+^ T cells [28–31]. Interestingly, despite these strong cytotoxic lymphocyte responses, analyses of biopsies from deceased patients reveal intact infected endothelial cells [32]. Recently, hantaviruses where shown to inhibit cytotoxic lymphocyte-mediated killing by interfering with their capacity to induce apoptosis in infected target cells. Hantaviruses efficiently inhibit cytotoxic granule-dependent induction of apoptosis by inhibiting granzyme B, and TRAIL-mediated induction of apoptosis by downregulating cell surface expression of DR5 [33–36]. The strong cytotoxic lymphocyte responses, combined with their inability to kill hantavirus-infected target cells, may contribute to the HFRS/HPS pathogenesis by triggering inflammatory responses, in a similar manner as, e.g., observed in familiar forms of hemophagocytic lymphohistiocytosis [37].

In addition to inhibiting cytotoxic lymphocyte-mediated killing of infected cells, we recently also showed that hantaviruses inhibit chemically induced apoptosis [33, 35]. Staurosporine (STS) is a pro-apoptotic chemical that induces intrinsic apoptosis [38]. In STS-exposed hantavirus-infected cells, the cleavage of procaspase-3 into active caspase-3 is hampered, suggesting an anti-apoptotic effect of hantaviruses upstream of caspase-3 [33, 35]. However, this implies that hantaviruses are equipped with additional unknown anti-apoptotic mechanisms, as neither inhibition of granzyme B nor downregulation of DR5 can explain inhibition of STS-induced apoptosis.

In the present study, we aimed at defining the mechanism modulated by hantaviruses to hinder STS-induced apoptosis upstream of the executioner caspase-3. We report that two different hantaviruses, ANDV and HTNV, inhibit activation of caspase-8 and -9, and inhibit the release of cyt C from mitochondria to the cytosol in infected cells. MOMP was not activated in infected cells, and we show that hantavirus induces the expression of the pro-survival factor BCL-2 in infected, but not in uninfected bystander, cells. Strikingly, silencing of BCL-2 or inhibition of its activity by the BCL-2 inhibitor ABT-737 sensitized ANDV- and HTNV-infected cells to STS-induced apoptosis, showing that the virus-induced up-regulation of BCL-2 is necessary for hantavirus-mediated inhibition of intrinsic apoptosis.

## Results

### Hantavirus inhibits activation of caspase -8 and -9

Hantaviruses inhibit STS-induced apoptosis, and less active caspase-3 is detected in hantavirus-infected cells [33, 35] explaining why infected cells do not undergo chemically induced apoptosis. However, how hantaviruses specifically inhibit intrinsic apoptosis is not known.

**Fig 1:**
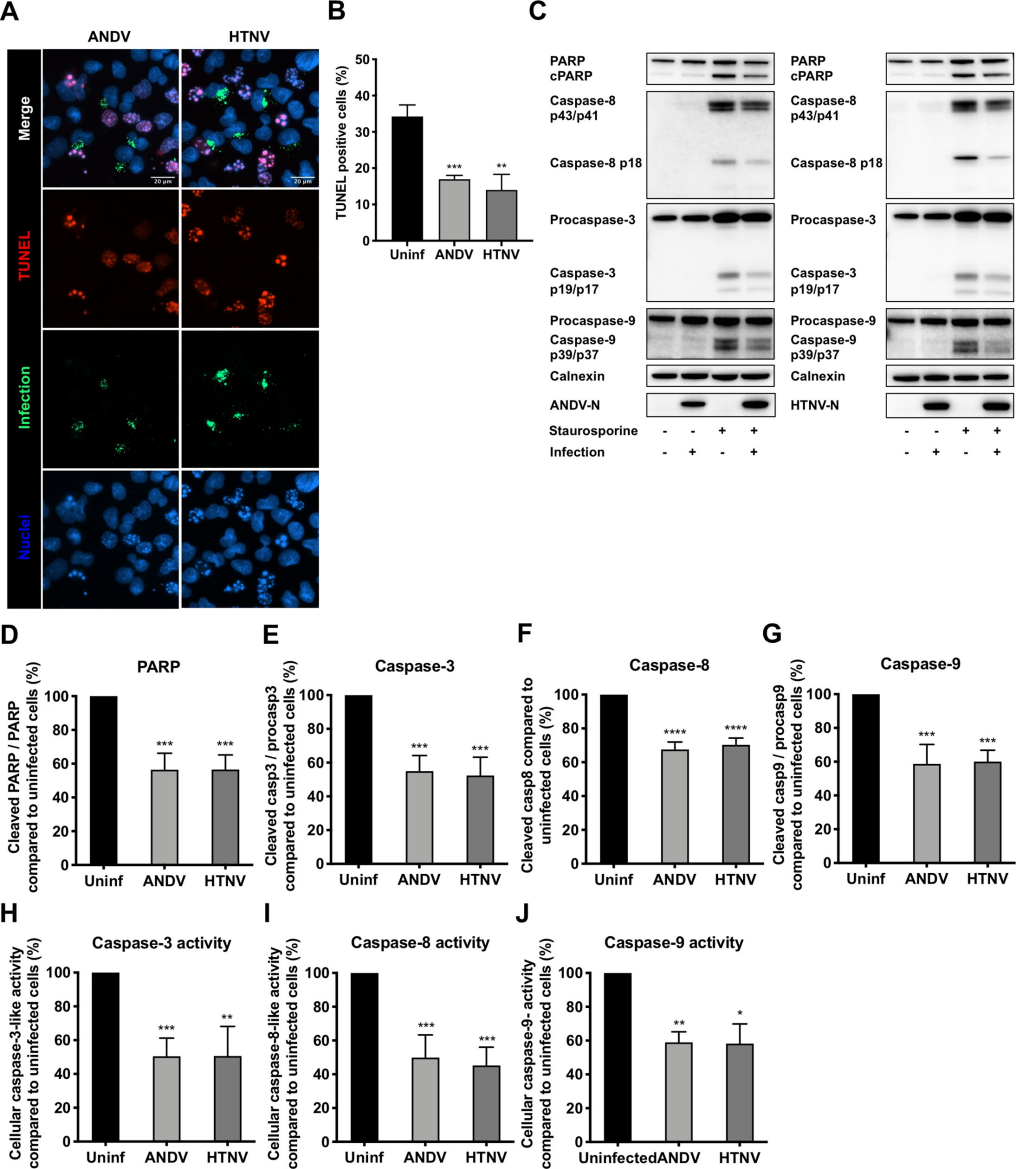
Hantavirus infection hampers the activation of caspases in staurosporine-exposed A549 cells. A549 cells were infected at a multiplicity of infection (MOI) of 1 in order to achieve ≥90% infection rates at 72 hours post-infection (see S1 Fig), when cells were treated with 2 μM of STS for 4 hours. For TUNEL analysis only, cells were infected at a lower MOI of 0.01 to achieve around 30% infection rates at 72 hours post-infection when cells were exposed to STS. **(A)** Representative immunofluorescence staining of ANDV-infected, HTNV-infected and uninfected cells after STS-treatment. TUNEL (red) was used to label apoptotic cells and polyclonal antibodies from convalescent patient serum (green) to detect ANDV- and HTNV-infected cells. DAPI (blue) was used for nuclear counterstaining. One representative experiment out of three independent experiments is shown. Scale bar, 20 μm. **(B)** Percentages of TUNEL positive cells after STS-treatment. Numbers of TUNEL-positive infected and uninfected cells on the same slides were determined using fluorescence microscopy. Data shown represent the mean ± SD from three independent experiments in which at least 300 cells were counted for each experiment. **(C)** Representative western blot of cleaved PARP, caspase-8, caspase-9 and caspase-3 from lysates of ANDV-infected, HTNV-infected and uninfected A549 cells after STS- treatment. ANDV and HTNV nucleocapsid protein (ANDV-N; HTNV-N) were detected using the monoclonal antibody 1C12. Calnexin was used as loading control. One representative experiment out of three independent experiments is shown. **(D-G)** Analysis of cleaved PARP (**D**), caspase-3 (**E**), caspase-8 (**F**) and caspase-9 (**G**) by band densitometry. Ratios between cleaved PARP and full-length PARP, between caspase-3 and procaspase-3, and between caspase-9 and procaspase-9, were respectively calculated and compared between infected and uninfected cells after STS-treatment. The amount of caspase-8 in infected cells was calculated and compared to that in uninfected cells after STS-treatment. STS-treated uninfected cells represent maximal PARP, procaspase-8, procaspase-3 or procaspase-9 cleavage at 4 hours after STS-treatment. Calnexin was used as loading control and band densitometry analysis was done using ImageJ. Data shown represent the mean ± SD from three independent experiments. **(H)** Graph displaying caspase-3 activity in hantavirus infected and uninfected cells after STS-treatment. Caspase-3 activity in uninfected cells represents maximal caspase-3 activity. Data shown represent the mean ± SD from three independent experiments. **(I)** Graph displaying caspase-8 activity in hantavirus infected and uninfected cells after STS-treatment. Caspase-8 activity in uninfected cells represents maximal caspase-8 activity. Data shown represent the mean ± SD from three independent experiments. **(J)** Graph displaying caspase-9 activity in hantavirus infected and uninfected cells after STS-treatment. Caspase-9 activity in uninfected cells represents maximal caspase-9 activity. Data shown represent the mean ± SD from three independent experiments. Paired t test was used for statistical evaluation: **** p<0.0001, *** p<0.001, ** p<0.01, * p<0.05.

To analyze for the mechanism used by hantaviruses to inhibit apoptosis, A549 cells were infected with either ANDV or HTNV, or left uninfected. At 72 hours post-infection (p.i.), the cells were exposed to STS for 4 hours. As earlier reported [33, 35], we observed less TUNEL-positive infected, compared to uninfected, STS-treated cells (Fig 1A and 1B). Also, infected STS-treated cells showed less cleaved poly ADP-ribose polymerase (PARP) and activated caspase-3 compared to uninfected STS-treated cells (Fig 1C–1E). STS-treated ANDV-infected cells showed 44 ± 9.7% less cleaved PARP and 45 ± 9.3% less cleaved, activated, caspase-3, and HTNV-infected cells showed 43 ± 8.6% less cleaved PARP and 48 ± 10.8% less cleaved, activated, caspase-3, as compared to uninfected cells (Fig 1D and 1E).

Activated caspase-8 and caspase-9 cleave, thereby activating, effector caspases, predominantly caspase-3, which results in subsequent apoptotic cell death. We therefore also analyzed for caspase-8 and caspase-9 activation. The cleavage of both procaspase-8 and procaspase-9 into their active forms was clearly hampered in STS-treated hantavirus-infected, compared to uninfected, cells (Fig 1C and 1F and 1G). Compared to STS-treated uninfected cells, 32 ± 4.4% and 30 ± 4.0% less cleaved caspase-8, and 41 ± 12% and 40 ± 6.8% less cleaved caspase-9, was observed for STS-treated ANDV-infected and HTNV-infected cells, respectively (Fig 1F and 1G). Moreover, analysis of the enzymatic activity of these caspases revealed decreased caspase-3 activity (Fig 1H), caspase-8 activity (Fig 1I), and caspase-9 activity (Fig 1J) in STS-treated hantavirus-infected, compared to uninfected, cells. Taken together, these data show that activation of caspase-8 and caspase-9 is hampered in hantavirus-infected cells.

### Hantavirus inhibits STS-mediated release of cytochrome C

**Fig 2:**
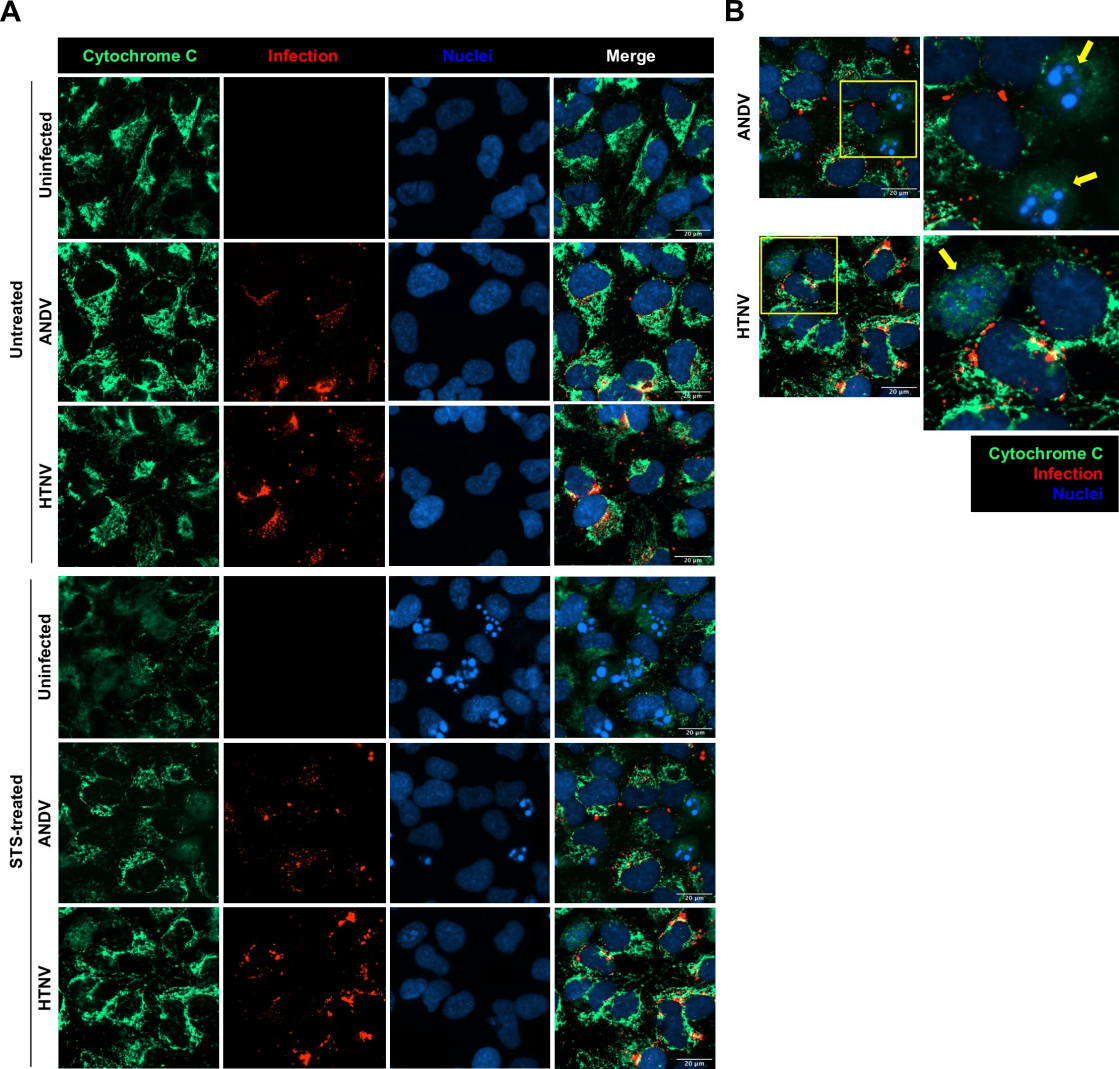
Hantavirus infection inhibits STS-mediated release of cytochrome C. A549 cells were infected at MOI 1 and treated with STS for 4 hours at 72 hours p.i. **(A)** Immunofluorescence images showing cytochrome C (green) in ANDV- and HTNV-infected cells (red) after exposure to STS. DAPI (blue) was used for nuclear counterstaining. One representative experiment out of three independent experiments is shown. Scale bar, 20 μm. **(B)** Magnified immunofluorescence pictures showing cytochrome C (green) release in bystander uninfected cells (yellow arrows) but not in ANDV- and HTNV-infected cells (red). DAPI (blue) was used for nuclear counterstaining. Yellow arrows indicate bystander uninfected cells. One representative experiment out of three independent experiments is shown. Scale bar, 20 μm.

STS induces mitochondrial depolarization, thereby triggering cyt C release, which subsequently results in the activation of apoptosis-inducing pathways, including caspase-9 activation [17]. Therefore, we next analyzed if hantaviruses could inhibit the release of cyt C from mitochondria. As expected, mitochondrial release of cyt C was clearly observed in STS-treated uninfected cells (Fig 2A). However, in ANDV-infected and HTNV-infected cells, cyt C release was significantly impaired, with most cyt C remaining in mitochondria of the infected cells (Fig 2A). Further, when analyzing infected and uninfected cells on the same slides, cyt C release was detected in uninfected bystander cells (Fig 2B), suggesting that the inhibition of cyt C release specifically occurred in infected cells.

### Hantavirus inhibits STS-mediated mitochondrial membrane permeabilization and activation of BAX

**Fig 3:**
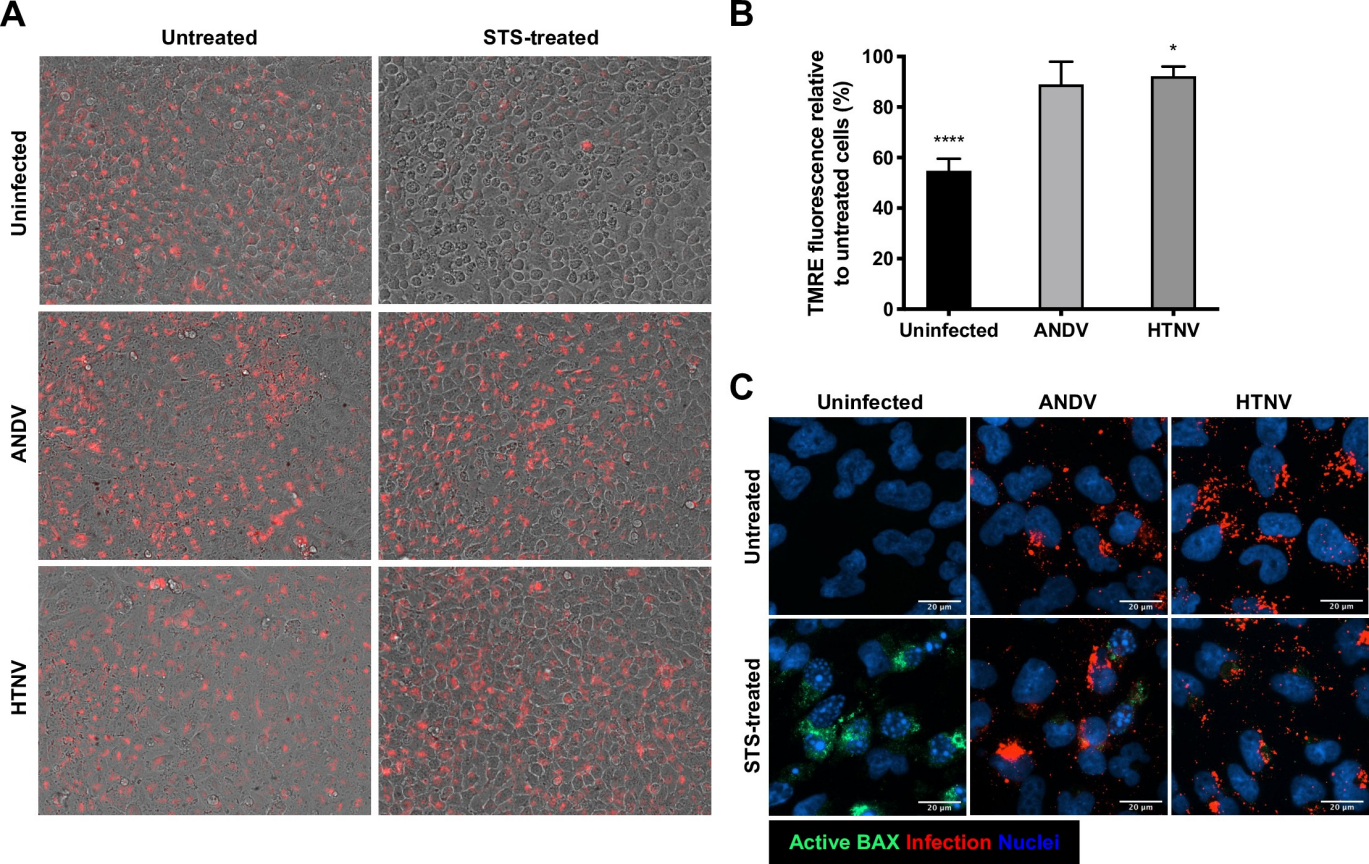
Hantavirus infection hinders mitochondrial membrane potential loss in cells after exposure to STS. A549 cells were infected at MOI 1 and treated with STS for 4 hours at 72 hours p.i. **(A)** TMRE staining (red) was performed in order to measure the mitochondrial transmembrane potential in ANDV-infected, HTNV-infected and uninfected A549 cells after STS-treatment. Images represent the overlay of bright field images with the corresponding TMRE fluorescence images. One representative experiment out of three independent experiments is shown. **(B)** Graph displaying as percentage the reduction in TMRE fluorescence signal in STS- treated ANDV-infected, HTNV-infected and uninfected cells compared to the TMRE fluorescence in untreated cells. Data shown represent the mean ± SD from three independent experiments. **(C)** Representative immunofluorescence images showing activation of BAX after STS-treatment. Active BAX (green) was observed in uninfected cells after exposure to STS but not in hantavirus infected (red) cells. DAPI (blue) was used for nuclear counterstaining. One representative experiment out of three independent experiments is shown. Scale bar, 20 μm. Paired t test was used for statistical evaluation: **** p<0.0001, * p<0.05.

The observation that hantavirus-infected cells do not release cyt C into the cytosol upon STS-treatment (Fig 2) suggested that hantaviruses prevent mitochondrial depolarization thereby preventing release of cyt C. To analyze if hantaviruses could inhibit STS-mediated changes in mitochondrial permeabilization, cells were loaded with the cell permeable dye tetramethylrhodamine-ethyl ester (TMRE) that accumulates in viable, active mitochondria [39, 40]. TMRE accumulation was observed in untreated hantavirus-infected and uninfected cells (Fig 3A). Upon treatment with STS, the TMRE fluorescence signal clearly decreased in uninfected cells whereas in hantavirus-infected cells it remained intense in the majority of the cells (Fig 3A). Overall, STS-treatment caused a 45 ± 4.7% reduction in TMRE fluorescence in uninfected cells, compared to a 7.0 ± 15% and 7.8 ± 3.9% reduction in ANDV- and HTNV-infected cells, respectively (Fig 3B). BAX and other pro-apoptotic BCL-2 family members oligomerize and form pores at the mitochondrial membrane, needed for release of cyt C from mitochondria [19, 20]. Staining for active BAX showed an overall activation of BAX in STS-treated uninfected cells (Fig 3C). In contrast, little active BAX was detected in ANDV- and HTNV-infected STS-treated cells (Fig 3C). Overall, these results indicate a strong effect of hantavirus infection on the mitochondrial intrinsic pathway toward apoptosis by hindering the permeabilization of mitochondria and the subsequent release of cyt C.

### Hantavirus induces BCL-2 expression in infected A549 cells

**Fig 4:**
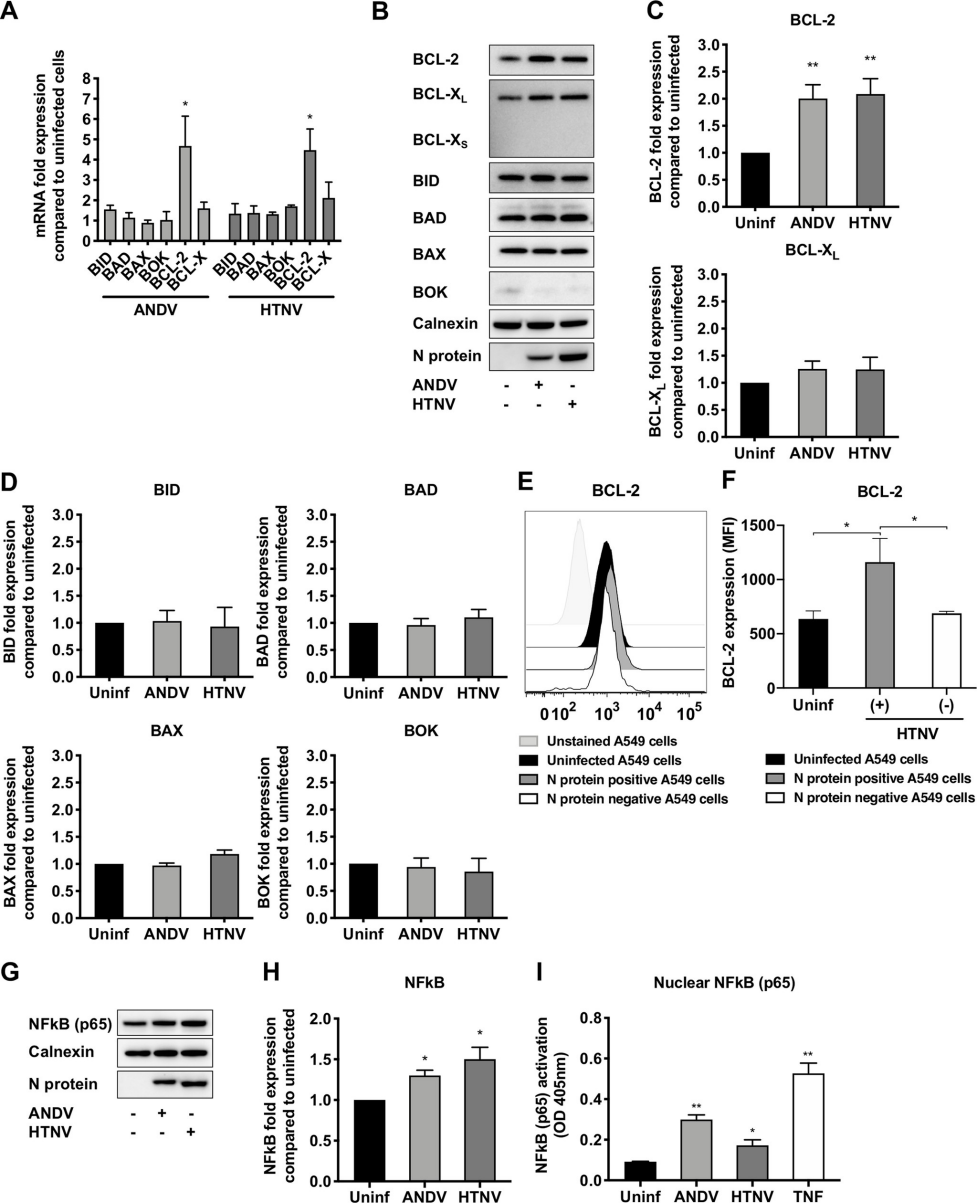
Hantavirus infection causes increased expression of the pro-survival factor BCL-2 and induces NFkB (p65) activation. A549 cells were infected with ANDV or HTNV at MOI 1. At 72 hours p.i. the expression of pro- and anti-apoptotic BCL-2 family members were determined. **(A)** Graph displaying mRNA expression levels of the pro-apoptotic BCL-2 family members BID, BAD, BAX and BOK, and of the pro-survival factors BCL-2 and BCL-X, in ANDV- and HTNV-infected cells. Data shown represent the mean ± SD from three independent experiments **(B)** Representative western blot of BCL-2, BCL-X_L_ and BCL-X_S_, BID, BAD, BAX and BOK from lysates of ANDV-infected, HTNV-infected and uninfected A549 cells. ANDV and HTNV nucleocapsid protein (ANDV-N; HTNV-N) were stained using the monoclonal antibody 1C12. Calnexin was used as loading control. One representative experiment out of three independent experiments is shown. **(C)** Fold change in total cellular BCL-2 and BCL-X_L_ in ANDV- and HTNV-infected cells compared to uninfected cells. Expression was measured by band densitometry analysis using the software ImageJ and calculated as fold change increase compared to total cellular BCL-2 and BCL-X_L_ in uninfected cells at 72 hours p.i. Calnexin was used as loading control. Data shown represent the mean ± SD from three independent experiments. **(D)** Fold change in total cellular BID, BAD, BAX and BOK in ANDV- and HTNV-infected cells compared to uninfected cells. Expression was measured by band densitometry analysis using the software ImageJ and calculated as fold change increase compared to total cellular BID, BAD, BAX and BOK in uninfected cells at 72 hours p.i. Calnexin was used as loading control. Data shown represent the mean ± SD from three independent experiments. **(E)** Representative histogram of flow cytometry analysis of BCL-2 expression in infected (N-protein^+^) and uninfected (N-protein^-^) cells within the HTNV-exposed cells, as well as in uninfected cells. One representative experiment out of three independent experiments is shown. **(F)** Flow cytometry analysis of BCL-2 shown as mean fluorescence intensity (MFI) after gating on N-protein^+^ and N-protein^-^ cells within the HTNV-infected cells condition, as well as on uninfected cells. Data shown represent the mean ± SD from three independent experiments. **(G)** Representative western blot of NFkB (p65) from lysates of ANDV-infected, HTNV-infected and uninfected cells. ANDV and HTNV nucleocapsid protein (ANDV-N; HTNV-N) were visualized using the monoclonal antibody 1C12. Calnexin was used as loading control. One representative experiment out of three independent experiments is shown. **(H)** Fold change in total cellular NFkB (p65) in ANDV- and HTNV-infected cells compared to uninfected cells. Expression was measured by band densitometry analysis using the software ImageJ and calculated as fold change increase compared to total cellular NFkB (p65) in uninfected cells at 72 hours p.i. Calnexin was used as loading control. Data shown represent the mean ± SD from three independent experiments. **(I)** NFkB (p65) activation analyzed from nuclear extracts obtained from ANDV-infected, HTNV-infected and uninfected cells. TNF-α-stimulated cells were used as positive controls for NFkB (p65) nuclear translocation. Total protein content in samples was quantified before analysis of NFkB (p65) activation. Data shown represent the mean ± SD from three independent experiments. Paired t test was used for statistical evaluation: ** p<0.01, * p<0.05.

Activation of MOMP and mitochondrial depolarization can be inhibited by pro-survival BCL-2 family members, especially BCL-2 [38, 41, 42]. Therefore, we next analyzed if hantaviruses could affect the expression of different BCL-2 family members. Indeed, analysis of the mRNA levels of different BCL-2 family members revealed a significant increase in BCL-2 mRNA levels in hantavirus-infected cells (Fig 4A). ANDV-infected cells showed a 4.7 ± 1.5 (mean ± SD) fold increase and HTNV-infected cells a 4.5 ± 1.0-fold increase in BCL-2 mRNA expression compared to uninfected cells. Another pro-survival BCL-2 family member, BCL-X_L_, was slightly up-regulated in HTNV-infected cells (2.2 ± 0.6-fold increase) compared to uninfected cells (Fig 4A). In contrast, none of the pro-apoptotic BCL-2 family members BID, BAD, BAX and BOK were significantly up-regulated in infected cells as assessed at the mRNA level (Fig 4A). Analysis of protein levels by immunoblotting revealed likewise clearly increased levels of BCL-2 in hantavirus-infected cells (Fig 4B and 4C). However, no significant increase in BCL-X_L_ expression was detected in hantavirus-infected cells (Fig 4B and 4C), and levels of the pro-apoptotic BCL-2 family members BID, BAD, BAX and BOK did not clearly differ between infected and uninfected cells (Fig 4B and 4D). Flow cytometry analysis confirmed hantavirus-induced BCL-2 expression (Fig 4E and 4F), and further revealed that infected (N protein^+^), but not uninfected (N protein^-^), HTNV-exposed cells showed increased BCL-2 expression (Fig 4E and 4F). The finding that only infected cells, and not uninfected bystander cells, showed increased levels of BCL-2 indicated a possible role for hantavirus proteins in activating BCL-2 production. To test this, we transfected cells with plasmids expressing hantavirus N protein or the glycoproteins Gn/Gc, and then analyzed for levels of BCL-2. However, neither expression of hantavirus N protein nor expression of Gn/Gc resulted in increased BCL-2 expression, as determined by western blot as well as by flow cytometry analyses after gating on N protein positive and glycoprotein positive cells (S2 Fig).

BCL-2 has been shown to be transcriptionally regulated by nuclear factor-kappa B (NFkB) [43–45]. We therefore analyzed if hantavirus infection could induce NFkB expression and nuclear translocation. The levels of total cellular NFkB (p65) were slightly increased in ANDV- and HTNV-infected A549 cells (Fig 4G and 4H). Moreover, we observed increased levels of NFkB (p65) in the nucleus in ANDV- and HTNV-infected cells compared to uninfected cells (Fig 4I), suggesting that hantavirus infection triggers NFkB activation, potentially contributing to the observed increased transcription of BCL-2 mRNA (Fig 4A).

### Silencing of BCL-2 sensitizes hantavirus-infected cells to apoptosis

**Fig 5:**
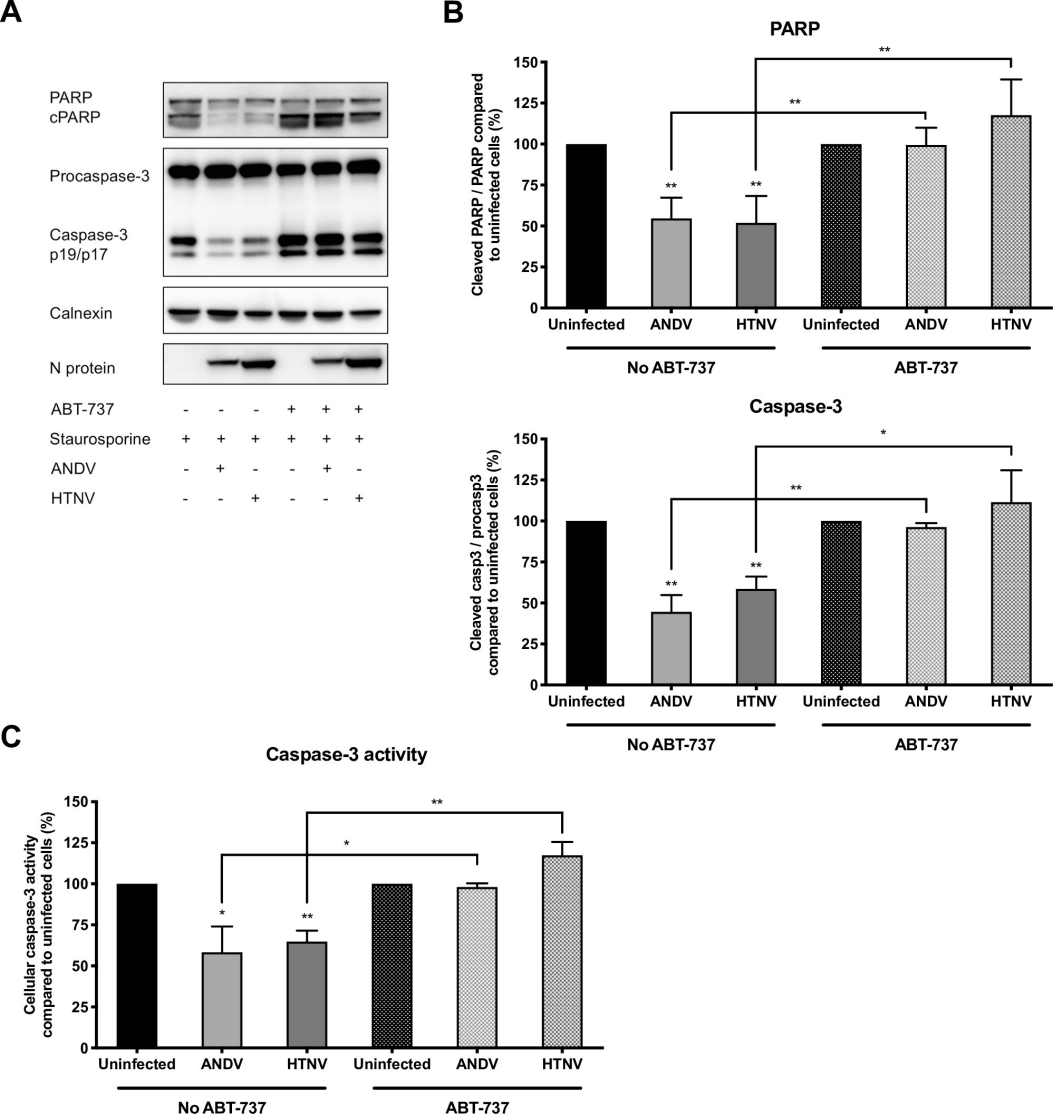
BCL-2 inhibition results in apoptosis of STS-treated hantavirus-infected cells. A549 cells were infected with ANDV or HTNV at MOI 1. Cells were exposed to ABT-737 for 16 hours prior to STS-treatment at 72 hours p.i. After 4 hours of STS-treatment, cells were harvested for analysis of cleaved PARP and caspase-3 cleavage and activity. **(A)** Representative western blot of cleaved PARP and caspase-3 from lysates of ANDV-infected, HTNV-infected and uninfected A549 cells after ABT-737 treatment and exposure to STS. ANDV and HTNV nucleocapsid protein (ANDV-N; HTNV-N) were visualized using the monoclonal antibody 1C12. Calnexin was used as loading control. One representative experiment out of three independent experiments is shown. **(B)** Analysis of PARP and caspase-3 cleavage as a ratio between cleaved PARP and full-length PARP and between caspase-3 and procaspase-3. Ratios were calculated and compared between infected and uninfected cells after ABT-737 and STS-treatment. STS-treated, and ABT-737- plus STS-treated, uninfected cells represent maximal PARP and caspase-3 cleavage at 4 hours after STS-treatment. Calnexin was used as loading control and band densitometry analysis was done using ImageJ. Data shown represent the mean ± SD from three independent experiments. **(C)** Graph displaying caspase-3 activity in hantavirus infected and uninfected cells after STS-treatment. Caspase-3 activity in uninfected cells represents maximal caspase-3 activity. Data shown represent the mean ± SD from three independent experiments. Paired t test was used for statistical evaluation: ** p<0.01, * p<0.05.

The finding that hantaviruses trigger the expression of the pro-survival factor BCL-2 in infected cells indicates a possible important, and specific, role for BCL-2 in hantavirus-mediated resistance to apoptosis. To further understand the potential implications of BCL-2 in infected cells, ANDV- and HTNV-infected cells were treated with the BCL-2 inhibitor ABT-737 before being exposed to STS. ABT-737-treated ANDV- and HTNV-infected cells were susceptible to STS-treatment, showing similar levels of cleaved PARP and activated caspase-3 (Fig 5A and 5B), and increased caspase-3 activity (Fig 5C), as observed for uninfected cells. ABT-737 treatment did not affect expression levels of BCL-2, BCL-X_L_, BID, BAD, BAX or BOK in treated cells (S3 Fig), suggesting that the observed effect was mainly caused by inhibition of BCL-2.

**Fig 6:**
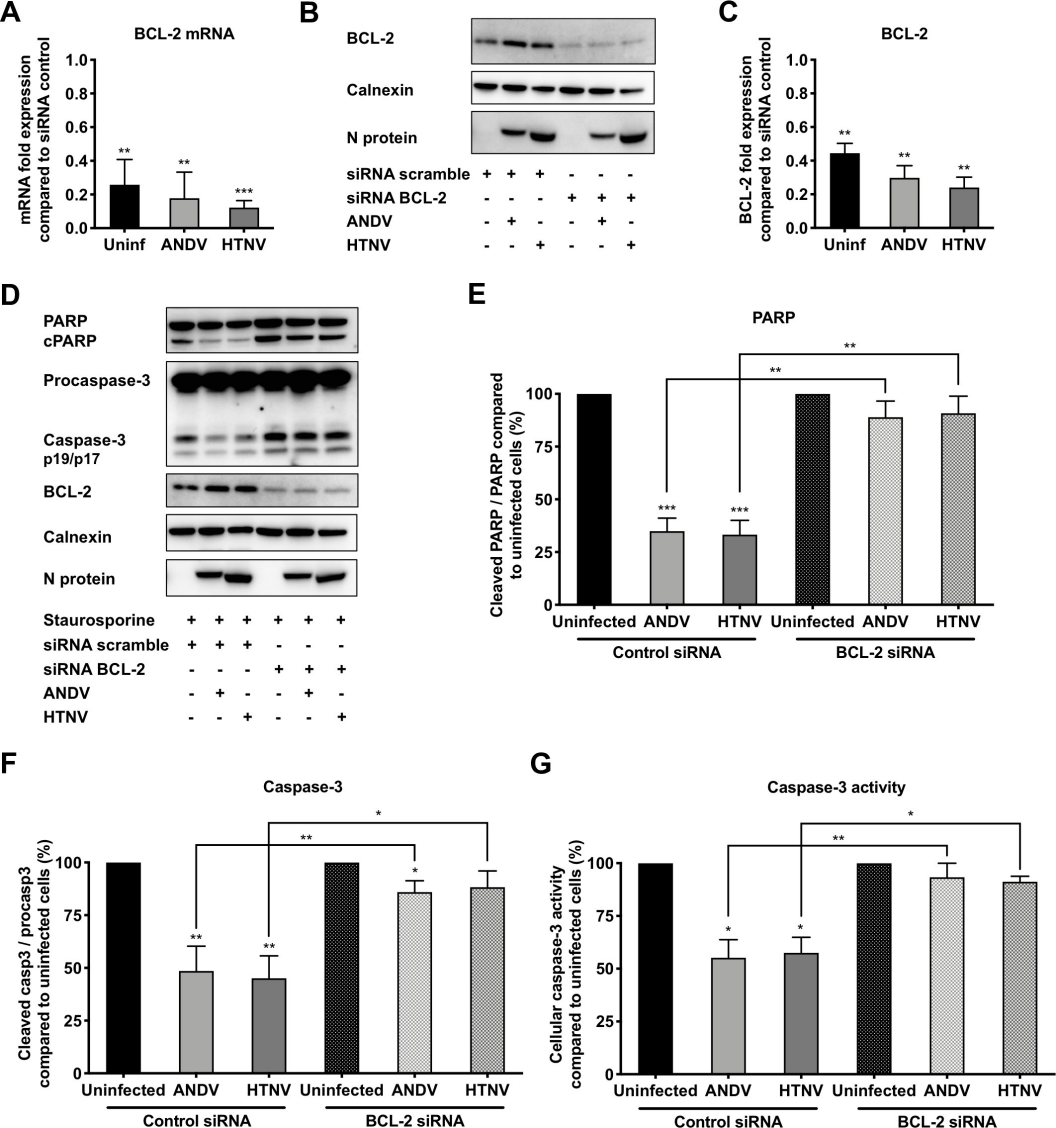
Silencing of BCL-2 renders hantavirus-infected cells vulnerable to STS-mediated apoptosis. A549 cells were infected with ANDV or HTNV at MOI 1, and at 24 hours p.i. the cells were transfected with siRNA targeting BCL-2. Cells were exposed to STS at 72 hours p.i. and harvested for analysis after 4 hours of STS-treatment. **(A)** Graph displaying mRNA expression levels of BCL-2 as fold change in ANDV-infected, HTNV-infected and uninfected cells after BCL-2 knock down by siRNA transfection, compared to BCL-2 mRNA expression in control siRNA scramble-transfected cells. Data shown represent the mean ± SD from three independent experiments. **(B)** Representative western blot of BCL-2 silencing from lysates of ANDV-infected, HTNV-infected, and uninfected A549 cells after BCL-2 knock down. siRNA scramble transfected cells were used as control. ANDV and HTNV nucleocapsid protein (ANDV-N; HTNV-N) were visualized using the monoclonal antibody 1C12. Calnexin was used as loading control. One representative experiment out of three independent experiments is shown. **(C)** Fold change in levels of total cellular BCL-2 in ANDV-infected, HTNV-infected and uninfected cells after BCL-2 knock down. Expression was measured by band densitometry analysis using the software ImageJ and calculated as fold change increase compared to BCL-2 expression in control siRNA scramble-transfected cells. Calnexin was used as loading control. Data shown represent the mean ± SD from three independent experiments. **(D)** Representative western blot of cleaved PARP and caspase-3 from lysates of ANDV-infected, HTNV-infected and uninfected A549 cells after BCL-2 knock down and STS-treatment. ANDV and HTNV nucleocapsid protein (ANDV-N; HTNV-N) were detected using the monoclonal antibody 1C12. Calnexin was used as loading control. One representative experiment out of three independent experiments is shown. **(E-F)** Analysis of PARP and caspase-3 cleavage as a ratio between cleaved PARP and full-length PARP (**E**) and between caspase-3 and procaspase-3 (**F**). Ratios were calculated and compared between infected and uninfected cells after BCL-2 knock down and STS-treatment. STS-treated, and BCL-2-silenced plus STS-treated, uninfected cells represent maximal PARP and caspase-3 cleavage at 4 hours after STS-treatment. Calnexin was used as loading control and band densitometry analysis was done using ImageJ. Data shown represent the mean ± SD from three independent experiments. **(G)** Graph displaying caspase-3 activity in hantavirus infected and uninfected cells after STS-treatment. Caspase-3 activity in uninfected cells represents maximal caspase-3 activity. Data shown represent the mean ± SD from three independent experiments. Paired t test was used for statistical evaluation: *** p<0.001, ** p<0.01, * p<0.05.

Taken together, these findings suggested that hantaviruses are dependent on BCL-2 to inhibit intrinsic apoptosis. To further test this, we knocked down BCL-2 expression using siRNA (Fig 6A–6C), and then exposed these infected and uninfected cells to STS. Knock down of BCL-2 resulted in increased PARP cleavage (Fig 6D and 6E) and increased levels of active, cleaved, caspase-3 (Fig 6D and 6F) in STS-treated hantavirus-infected cells. In line with these results, BCL-2 knock down also resulted in increased caspase-3 activity in STS-treated hantavirus-infected cells; similar levels of caspase-3 activity were observed in infected and uninfected STS-treated BCL-2 knock down cells (Fig 6G). No clear effect on progeny virus production was detected in supernatants from hantavirus-infected cells when inhibiting BCL-2 by ABT-737 as well as when silencing the expression of BCL-2 by siRNA, compared to the progeny virus detected in untreated hantavirus-infected cells (S4 Fig). Taken together, these results suggest that BCL-2 is crucial for hantavirus-mediated resistance to apoptosis but is not required for hantavirus replication.

### Hantaviruses induce BCL-2 expression in primary endothelial cells

**Fig 7:**
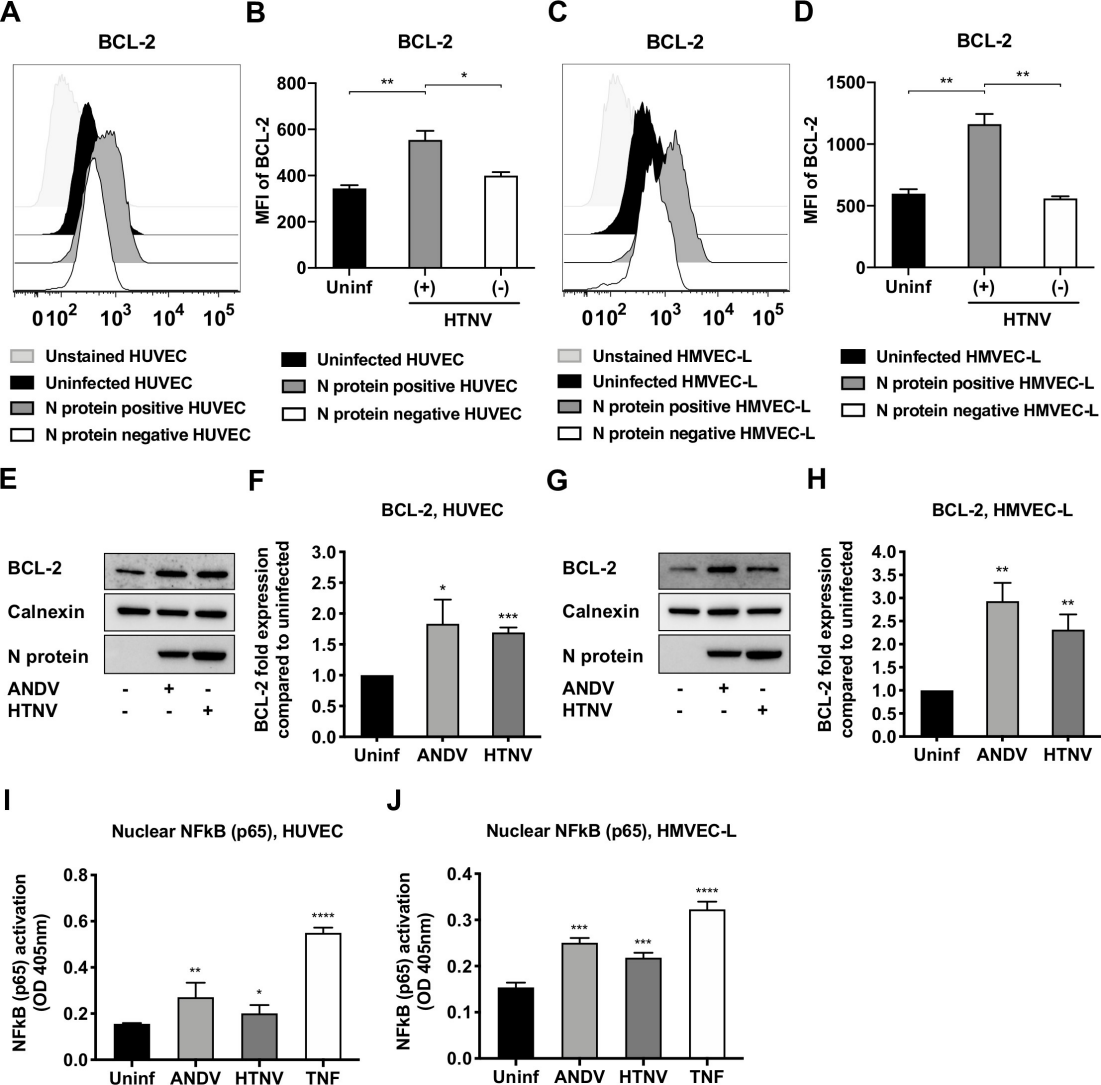
The expression of the pro-survival factor BCL-2 is increased in hantavirus-infected endothelial cells. HUVECs and HMVECs were infected with ANDV or HTNV at MOI 1. At 72 hours p.i. the expression of the anti-apoptotic BCL-2 and the nuclear translocation of NFkB were determined. **(A)** Representative histogram of flow cytometry analysis of BCL-2 expression in infected (N-protein^+^) and uninfected (N-protein^-^) cells within the HTNV-exposed HUVECs condition, as well as in uninfected HUVECs. One representative experiment out of three independent experiments is shown. **(B)** Flow cytometry analysis of BCL-2 expression in HUVECs shown as mean fluorescence intensity (MFI) after gating on N-protein^+^ and N-protein^-^ cells within the HTNV-infected cells condition, as well as on uninfected cells. Data shown represent the mean ± SD from three independent experiments. **(C)** Representative histogram of flow cytometry analysis of BCL-2 expression in infected (N-protein^+^) and uninfected (N-protein^-^) cells within the HTNV-exposed HMVECs condition, as well as in uninfected HMVECs. One representative experiment out of three independent experiments is shown. **(D)** Flow cytometry analysis of BCL-2 expression in HMVECs shown as mean fluorescence intensity (MFI) after gating on N-protein^+^ and N-protein^-^ cells within the HTNV-infected cells condition, as well as on uninfected cells. Data shown represent the mean ± SD from three independent experiments. **(E)** Representative western blot of BCL-2 from lysates of ANDV-infected, HTNV-infected and uninfected HUVECs. ANDV and HTNV nucleocapsid protein (ANDV-N; HTNV-N) were detected using the monoclonal antibody 1C12. Calnexin was used as loading control. One representative experiment out of three independent experiments is shown. **(F)** Fold change in total cellular BCL-2 in ANDV- and HTNV-infected, compared to uninfected, HUVECs. Expression was measured by band densitometry analysis using the software ImageJ and calculated as fold change increase compared to total cellular BCL-2 in uninfected cells at 72 hours p.i. Calnexin was used as loading control. Data shown represent the mean ± SD from three independent experiments. **(G)** Representative western blot of BCL-2 from lysates of ANDV-infected, HTNV-infected and uninfected HMVECs. ANDV and HTNV nucleocapsid protein (ANDV-N; HTNV-N) were visualized using the monoclonal antibody 1C12. Calnexin was used as loading control. One representative experiment out of three independent experiments is shown. **(H)** Fold change in total cellular BCL-2 in ANDV- and HTNV-infected, compared to uninfected, HMVECs. Expression was measured by band densitometry analysis using the software ImageJ and calculated as fold change increase compared to total cellular BCL-2 in uninfected cells at 72 hours p.i. Calnexin was used as loading control. Data shown represent the mean ± SD from three independent experiments. **(I-J)** NFkB (p65) activation analyzed from nuclear extracts obtained from ANDV-infected, HTNV-infected and uninfected HUVECs and HMVECs. TNF-α-stimulated HUVECs and HMVECs were used as positive controls for NFkB (p65) nuclear translocation. Total protein content in samples was quantified before analysis of NFkB (p65) activation. Data shown represent the mean ± SD from three independent experiments. Paired t test was used for statistical evaluation: **** p<0.0001, *** p<0.001, ** p<0.01, * p<0.05.

Endothelial cells represent the main targets for hantaviruses [46]. Therefore, we sought to determine if ANDV and HTNV induced BCL-2 expression also in primary endothelial cells. To this end, primary human umbilical vein endothelial cells (HUVEC) and human lung microvascular endothelial cells (HMVEC) were infected with ANDV, HTNV, or left uninfected. At 72 hours p.i., cells were harvested for analysis of BCL-2 expression by flow cytometry and by western blot analyses. HTNV-infected (N protein^+^) cells revealed an increase in BCL-2 protein expression in both HUVECs and HMVECs (Fig 7A–7D). As observed in A549 cells (Fig 4E and 4F), only HTNV-infected cells, and not bystander uninfected cells, up-regulated BCL-2 (Fig 7A–7D). Further, examination of cell lysates from HUVECs and HMVECs confirmed a significant increase in BCL-2 in ANDV- and HTNV-infected, compared to uninfected, cells (Fig 7E–7H). Finally, we examined the levels of NFkB (p65) translocation to the nucleus of hantavirus infected and uninfected HUVECs and HMVECs. As observed in A549 cells, higher levels of nuclear NFkB (p65) in ANDV- and HTNV-infected, compared to uninfected, HUVECs and HMVECs were observed (Fig 7I and 7J).

Overall, these results showed that both ANDV and HTNV induce BCL-2 up-regulation in infected primary endothelial cells. Together with our findings in A549 cells, these data indicate that overexpression of BCL-2 represents a common effect induced by hantaviruses in infected cells.

## Discussion

Hantaviruses have a strong anti-apoptotic potential. This is revealed by their ability to inhibit cytotoxic granule-dependent induction of apoptosis in infected cells via inhibition of granzyme B [33], and downregulation of cell surface expression of DR5 [36]. In addition to inhibiting cytotoxic lymphocyte-mediated killing of infected cells, we recently showed that hantaviruses also inhibit intrinsic apoptosis [33, 35]. Importantly, intrinsic apoptosis, e.g., induced by STS, is dependent on other signaling pathways than those used by cytotoxic lymphocytes to induce apoptosis in target cells, suggesting that hantaviruses are equipped with a third, specific, anti-apoptotic mechanism. Here we show that hantavirus infection leads to increased production of anti-apoptotic BCL-2, which protects mitochondria from MOMP and release of cyt C, thereby inhibiting intrinsic apoptosis.

Apoptosis is induced in many different ways and represents a tightly regulated defense mechanism by which replication and spread of viruses is limited. Consequently, through constant host-pathogen interactions, many viruses have evolved strategies to subvert the apoptotic machinery of a cell by interfering with one or more apoptosis-inducing pathway(s). The finding that hantaviruses can interfere with mitochondrial membrane permeabilization to block apoptosis mimic strategies used also by other viruses [47–49]. Several DNA viruses, such as Epstein-Barr virus (EBV), encode for viral homologues of BCL-2 [50]. Another example is Rubella virus, the capsid protein of which has been reported to attenuate the formation of pores in mitochondria, thus impeding the release of cyt C and downstream activation of caspase-3 [51, 52]. Viruses can also trigger cellular production of BCL-2. NFkB is activated during many viral infections [53] and mediates anti-apoptotic effects by inducing the expression of anti-apoptotic genes, including BCL-2 [43, 44, 54]. Hepatitis C virus (HCV) and Human T-cell leukemia virus (HTLV) both induce BCL-X_L_ expression, and HTLV also induces BCL-2 expression, involving the activation of NFkB [55–57]. In line with this, we observed increased activation of NFkB in hantavirus-infected cells, suggesting that it is involved in the observed induction of BCL-2. Importantly, we only observed increased BCL-2 expression in infected cells. However, no clear effect on BCL-2 expression was observed in cells expressing the N protein or the Gn/Gc proteins, and uninfected bystander cells showed similar levels as observed in cells not exposed to hantavirus, indicating that hantavirus infection *per se* is needed for upregulation of BCL-2.

Viral interference with host cell apoptosis not only leads to enhanced viral replication, thereby potentially contributing to pathogenesis, but may also promote cell transformation and tumour progression [58]. Several viruses that are known to activate BCL-2 production, such as EBV, HCV and HTLV, have oncogenic potential [59]. Interestingly, HFRS-patients show an increased risk for lymphoma development [60]. The observations here reported, together with recent findings showing that hantaviruses protect infected cells from cytotoxic lymphocyte-mediated killing [33–36], suggest that hantaviruses are equipped with several mechanisms that, directly or indirectly, may be involved in and/or promote carcinogenesis.

In summary, we here report that hantaviruses induce BCL-2 upregulation thereby inhibiting intrinsic apoptosis by preventing mitochondrial membrane potential loss and the release of cyt C. In turn, this blocks the processing and activation of caspases, consequently hampering the induction of apoptosis. This mechanism likely represents a crucial countermeasure displayed by hantaviruses to avert succumbing to the cell’s death machinery and may be involved in human hantavirus pathogenesis.

## Materials and methods

### Cells and virus stocks

The human lung epithelial cell line A549 (American Type Culture Collection (ATCC) CLL-185) was grown in MEM supplemented with 7.5% FBS, L-glutamine, 100 U/mL of penicillin, and 100 μg/mL of streptomycin (all from Thermo Fisher Scientific). Primary human umbilical vein endothelial cells (HUVEC) and primary lung microvascular endothelial cells (HMVEC) were from Lonza and grown in endothelial growth medium (EGM-2) supplemented with growth factors, according to the manufacturer’s instructions (Lonza). Vero E6 cells (ATCC Vero C1008) were maintained in MEM supplemented with 5% FCS, L-glutamine, 100 U/mL of penicillin, and 100 μg/mL of streptomycin in 5% CO_2_ at 37°C. Propagation and titration of the viruses ANDV strain Chile-9717869 and HTNV strain 76–118 [61], were performed on Vero E6 cells as earlier described [62]. Virus stocks were tested negative for *Mycoplasma sp*. as analyzed by DAPI staining and PCR detection kit (MicrosartRESEARCH Mycoplasma from Sartorius). All experiments involving live hantaviruses were conducted in a biosafety level-3 laboratory.

### Chemicals, antibodies and other reagents

Staurosporine (STS) was purchased from Biovision. Tetramethylrhodamine, Ethyl Ester, Perchlorate (TMRE) was from Thermo Fisher Scientific. The small molecule drug ABT-737 was from Active Biochem. 3′,5,5′-tetramethylbenzidine was from Sigma-Aldrich. For viral titrations, polyclonal monkey anti-PUUV serum and bank vole monoclonal antibody (mAb) 1C12 were used as primary antibodies [63]. The secondary antibodies horseradish peroxidase-conjugated goat anti-human IgG and horseradish peroxidase-conjugated goat anti-mouse IgG were from BioRad. For immunofluorescence, polyclonal antibodies (pAb) from convalescent patient sera were used to detect infected cells. The mAbs against Cytochrome C (6H2.B4, from Cell Signaling Technologies) and BAX (active monomer; 6A7, from Enzo Life Sciences) were used for immunofluorescence. The secondary antibodies anti-human IgG Alexa Fluor 594 conjugate, anti-human IgG Alexa Fluor 488 conjugate, and anti-mouse IgG Alexa Fluor 488 conjugate were all from Life Technologies. Nuclei were counter-stained with DAPI (Life Technologies). For western blotting, the following mAbs were used according to manufacturers’ recommendations: PARP (#9542), cleaved caspase-8 (#8592), caspase-9 (#9502), BID (#2002), BAD (#9292), BAX (#2774), BOK (#86875), NF-kB p65 (#8242) and calnexin (#2433) were all from Cell Signaling Technologies. The mAbs against caspase-3 (#ab13585) and against BCL-2 (#ab182858) were from Abcam. The mAb against BCL-X (#MAB4625) was from Merck-Millipore. The mAb 1C12, specific for detection of hantavirus nucleocapsid protein, was used as previously described [63], and the mAb 10B8 (#ab34763) from Abcam was used to detect hantavirus glycoprotein.

### Hantavirus infection of A549 cells

Infection was performed after diluting the virus stocks in Hanks’ balanced salt solution supplemented with 2% FBS, 0.02 M HEPES, 100 U/mL of penicillin, and 100 μg/mL of streptomycin (all from Thermo Fisher Scientific). When cells were 70% confluent, growth medium was removed from the cultures, the virus dilution was added, and cells were then incubated at 37°C with gentle shaking every 10 minutes. After 1 hour, the infectious medium was removed from the cultures and substituted with fresh, pre-warmed cell culture medium. For TUNEL analysis of cells exposed to STS, a low multiplicity of infection (MOI) of 0.01 was used resulting in around 20 to 30% of the cells being infected after 72 hours. Infectious work was otherwise performed at MOI 1, reaching infection rates ≥95% at 72 hours post-infection (S1 Fig).

### Virus titrations

Supernatants from infected cells were diluted 10-fold in Hank's balanced salt solution supplemented with 2% FBS, 0.02 M HEPES, 100 U/mL of penicillin, and 100 μg/mL of streptomycin (all from Thermo Fisher Scientific), and then added to confluent Vero E6 cells in 24-well plates for 1 hour. Cells were then overlaid with 0.5% agarose-medium (Eagles’ minimal essential medium (EMEM) containing 0.5% agarose, 5% FBS, 0.02 M HEPES, 100 U/mL of penicillin, and 100 μg/mL of streptomycin) and incubated for 7 days (HTNV) or 9 days (ANDV) at 37°C in 5% CO_2_. Agar medium was then removed, and cells fixed in methanol for 8 minutes at room temperature and then air-dried. Foci of infected cells were stained with polyclonal monkey anti-PUUV serum followed by horseradish peroxidase-conjugated goat anti-human IgG (Bio-Rad) (for ANDV), or with the bank vole mAb 1C12 followed by horseradish peroxidase-conjugated goat anti-mouse IgG (Bio-Rad) (for HTNV), and were visualized with 3,3′,5,5′-tetramethylbenzidine (Sigma-Aldrich) and counted.

### Staurosporine killing assay

STS was diluted in cell culture medium to a final concentration of 2 μM. Cells were incubated with the diluted chemical for 4 hours at 37°C, before being harvested for further analysis.

### ABT-737 treatment

When studying the effect of BCL-2 inhibition, A549 cells were exposed to the small molecule inhibitor ABT-737 for 16 hours prior to exposure to STS. ABT-737 was diluted in cell culture medium to a final concentration of 10 nM.

### Immunofluorescence

Cells grown on coverslips were fixed in 4% PFA for 20 minutes at room temperature (RT), or in methanol for 10 minutes at RT in the specific case of cytochrome C staining. Specimens were blocked in blocking solution (PBS containing 5% normal goat serum plus 0.3% Triton X-100) for 1 hour at RT. The samples were then incubated with primary antibodies for 1 hour at RT, washed three times with PBS and incubated with secondary antibodies for 1 hour at RT in the dark. Images were acquired with a fluorescent microscope (Nikon, Eclipse TE300). Image processing and quantification were performed with the software ImageJ (NIH; Imaging Processing and Analysis in Java; http://rsb.info.nih.gov/ij).

### TUNEL assay

A549 cells grown on coverslips were fixed with 4% PFA at RT for 20 minutes. The cells were then washed twice with PBS and permeabilized with 0.5% Triton X-100 in PBS for 8 minutes at 4°C. TUNEL (Terminal deoxynucleotidyl transferase mediated dUTP nick-end labeling) was performed as previously described [64], using an *in situ* cell death detection kit (TMR red from Roche). After the TUNEL chemical reaction, cells were stained for infection as described above and the nuclei counterstained with DAPI.

### TMRE assay

TMRE (Tetramethylrhodamine, ethyl ester) was used according to manufacturer’s (Thermo Fisher Scientific) instructions. In brief, cells were stained with pre-warmed medium containing 20 nM of TMRE. The cultures were incubated for 30 minutes at 37°C protected from light. Then the staining solution was removed and replaced by fresh pre-warmed PBS. The cells were subsequently analyzed by fluorescence imaging.

### Immunoblotting

Cells were collected and centrifuged at 1,500 rpm for 5 minutes at 4°C. Cells were then washed once with ice-cold PBS and cell pellets resuspended in lysis buffer (150 mM NaCL, 2 mM EDTA, 1% NP-40, and 50 mM Tris (pH 7.6)) complemented with protease and phosphatase inhibitors (cOmplete mini cocktail tablets and PhosSTOP inhibitor tablets from Roche), according to manufacturer’s guidelines. Samples were snap frozen until further analysis, or directly mixed 3:1 with NuPAGE LDS sample buffer (4x) (Thermo Fisher Scientific) supplemented with 2.5% 2-mercaptoethanol and incubated at 96°C for 10 minutes. The samples were run on NuPAGE Novex Bis-Tris protein gels (Thermo Fisher Scientific) and transferred to PVDF membranes using the iBlot2 Gel Transfer Device (Thermo Fisher Scientific). Blocking of the membranes was done at RT for 1 hour in PBS containing 5% nonfat powdered milk and 0.2% Tween 20 (Sigma-Aldrich). The membranes were then incubated with primary mAbs overnight at 4°C, followed by the addition of horseradish peroxidase-conjugated anti-mouse or anti-rabbit IgG (BioRad). Readout was obtained using ECL Plus Western blotting detection kit (GE Healthcare Life Sciences), following manufacturer’s instructions. Stripping of membranes was performed in Re-blot Plus mild antibody stripping solution, 10x (Merck Millipore), following the guidelines provided by the supplier. Band densitometry was analyzed using the software Image J (NIH; Imaging Processing and Analysis in Java; http://rsb.info.nih.gov/ij). Total protein concentration in samples was analyzed by Bradford assay and similar amounts of each sample were loaded to the gels.

### Flow cytometry

The mAb BCL-2 (#ab182858) from Abcam and the mAb B5D9 against HTNV nucleocapsid protein (#B5D9-POD) from ProGen were used for flow cytometry. Zenon antibody labeling kits against mouse IgG1 (Zenon AF488 Mouse IgG1 labeling kit) and rabbit IgG (Zenon AF594 Rabbit IgG labeling kit) were from Thermo Fisher Scientific. The LIVE/DEAD Fixable Aqua Dead Cell Stain Kit from Thermo Fisher Scientific was used in order to gate on live cells only. Staining of cells was performed as previously described [38]. Briefly, cells were harvested and washed once with FACS buffer (PBS containing 2% FBS and 2 mM EDTA). Cells were permeabilized for 30 minutes at RT in the dark with the Transcription Factor Permeabilization Buffer Set (Thermo Fisher Scientific) for subsequent intracellular staining. 10 μL of specific antibody against nucleocapsid protein or against BCL-2 were mixed with 2.5 μL Zenon labeling reagent (labeled Fab fragment) and incubated for 5 minutes at RT. Then, 2.5 μL of mix containing non-specific IgG was added. The mix was shortly vortexed and further incubated for another 5 minutes at RT. After permeabilization, the labeled antibodies were used for intracellular staining of cells for 20 minutes at RT in the dark. Stained cells were then washed twice with FACS buffer and fixed in 2% PFA for 10 minutes at RT in the dark. Samples were acquired on an LSR Fortessa (BD Biosciences) using FACSDiva software 8.0.1 (BD Biosciences) and analyzed with the software FlowJo version 9.8.1 (Tree Star Inc.).

### Enzymatic activity assays for caspases-3, -8 and -9

The enzymatic activity of caspase-3, caspase-8, and caspase-9 was assessed using specific colorimetric activity assays according to manufacturers’ instructions (Sigma-Aldrich for caspase-3 and caspase-8 activity assays, and Abcam for caspase-9 activity assay). For analysis of cellular caspase activity after STS treatment, cells were harvested and centrifuged at 1,500 rpm for 5 minutes at 4°C. The cells were washed once with ice-cold PBS. Cell pellets were re-suspended in lysis buffer without protease inhibitors and incubated for 15 minutes at 4°C. Cell lysates were centrifuged at 14,000 rpm for 10 minutes and supernatants snap frozen in liquid nitrogen. Total protein concentration in samples was analyzed by Bradford assay and similar amounts of each sample were used. The assays were performed at a total volume of 100 μL in 96 well plates following manufacturer’s instructions.

### Quantitative RT-PCR

Total RNA from harvested cells was isolated using TriPure isolation reagent (Roche). RNA was then treated with Turbo DNA-free (Ambion). cDNA synthesis was performed using the High-Capacity cDNA Reverse Transcription Kit and RNAse inhibitor from Thermo Fisher Scientific. TaqMan Gene Expression Assay for human BCL-2 (Hs00608023_m1), BCL-X (Hs00236329_m1), BID (Hs00609632_m1), BAD (Hs00188930_m1), BAX (Hs00751844_s1), BOK (Hs00261296_m1) and GAPDH (Hs02786624_g1), all from Thermo Fisher Scientific, were used. Quantitative RT-PCR was performed using the QuantStudio 5 Real-Time PCR instrument (Thermo Fisher Scientific). Obtained data were normalized to GAPDH and displayed as change in induction compared to that of uninfected cells.

### NFkB (p65) transcription factor activation assay

Nuclear extracts were obtained from harvested ANDV- and HTNV-infected and uninfected A549 cells using a nuclear extraction kit (#ab113474, Abcam), following manufacturer’s instructions. A549 cells stimulated with TNF (R&D systems) were used as positive controls for NFkB (p65) nuclear translocation. Nuclear extracts were snap frozen and stored at -80°C until analysis. Protein amount in samples was quantified before analysis of NFkB (p65) activation. Prepared nuclear extracts were tested for NFkB (p65) activity using the NFkB (p65) Transcription Factor Assay Kit (Colorimetric) from Abcam. In brief, nuclear extracts were added to the supplied plate pre-coated with single stranded DNA oligonucleotides containing NFkB (p65) consensus binding site. Samples were incubated for 1 hour at RT. Primary antibody was added after thorough washing of the wells and incubated for 1 hour at RT. The wells were washed, and HRP-conjugated secondary antibody added. The samples were then incubated for 1 hour at RT. Finally, the wells were washed and developing solution added for 2 to 10 minutes until a colored product was obtained. Stop solution was then added and absorbance measured at OD 450 nm with an ELISA reader.

### siRNA transfection

ON-TARGETplus SMARTpool siRNA Human BCL-2 (#L-003307-00-0010), ON-TARGETplus Non-targeting Control Pool (#D-001810-10-05), and the DharmaFECT transfection reagent were from Dharmacon. siRNA transfection was performed according to manufacturer’s instructions. In brief, the siRNA solutions were prepared in siRNA dilution buffer (Santa Cruz Biotechnology). The siRNA dilution and the DharmaFECT transfection reagent were further diluted in OptiMEM in two separate tubes and incubated for 5 minutes at RT. The contents of the tubes were mixed together and incubated for an extra 20 minutes at RT. Finally, the resulting siRNA transfection solution was added to the cultured cells.

### Expression of HTNV nucleocapsid protein and glycoproteins

A549 cells were transfected using Lipofectamine LTX and PLUS reagent diluted in Opti-MEM reduced serum medium (all from Thermo Fisher Scientific), according to manufacturer’s instructions. Plasmids expressing wild-type HTNV nucleocapsid protein (pCMV-Bios-HTNV-N), wild-type HTNV glycoproteins (pCMV-Bios-HTNV-G), and empty plasmid (pCMV-Bios-empty) were constructed by GenScript.

### Statistical methods

Paired t test was used for statistical analysis. The analysis was performed using GraphPad Prism software version 7.0 (GraphPad Software Inc.). Statistical significance is symbolized with asterisks (* p<0.05, ** p<0.01, *** p<0.001, **** p<0.0001).

## Supporting information

S1 Fig ANDV and HTNV infection rates in A549 cells. A549 cells were infected at MOI 1 for 72 hours.
**(A)** Immunofluorescence images of ANDV-infected, HTNV-infected and uninfected cells. One representative experiment out of three independent experiments is shown. Scale bar, 20 μm.
**(B)** Percentages of infected cells at 72 hours p.i. Numbers of infected and uninfected cells on the same slides were determined using fluorescence microscopy. Data shown represent the mean ± SD from three independent experiments in which at least 300 cells were counted for each experiment.
(TIF)

S2 Fig Single transfection of N or G proteins does not induce BCL-2. A549 cells were transfected with a plasmid expressing N protein, G proteins, or an empty vector as a control.
**(A)** Transfection rates of plasmids expressing N and G proteins. Cells were transfected and 24 hours later stained with antibodies specific for N (mAb 1C12) or Gn (mAb 10B8) proteins. Numbers of transfected cells were determined using fluorescence microscopy, and percentage of N expressing and Gn expressing cells calculated. Data shown represent the mean ± SD from three independent experiments in which at least 300 cells were counted for each experiment.
**(B)** mRNA expression levels of BCL-2 in N protein- and G protein-transfected cells compared to expression in empty plasmid-transfected cells. One representative experiment out of three independent experiments is shown.
**(C)** Representative western blot of BCL-2 expression from lysates of transfected cells stained with antibodies specific for N (mAb 1C12) or Gn (mAb 10B8) proteins. Calnexin was used as loading control. One representative experiment out of three independent experiments is shown.
**(D)** Fold change in total cellular BCL-2 in N or G expressing cells compared to cells transfected with an empty vector. Expression was measured by band densitometry analysis using the software ImageJ and calculated as fold change increase compared to total cellular BCL-2 in empty control cells. Calnexin was used as loading control. Data shown represent the mean ± SD from three independent experiments.
**(E)** Representative histogram of flow cytometry analysis of BCL-2 expression in transfected cells after gating on N-expressing or G-expressing cells. One representative experiment out of three independent experiments is shown.
**(F)** Flow cytometry analysis of BCL-2 shown as mean fluorescence intensity (MFI) in N-expressing or G-expressing cells compared to empty vector-transfected cells. Data shown represent the mean ± SD from three independent experiments.
(TIF)

S3 Fig ABT-737 does not alter the expression of BCL-2 family of proteins. A549 cells were infected with ANDV or HTNV at MOI 1. Approximately at 56 hours p.i., the cells were treated with ABT-737 for 16 hours. At 72 hours p.i. the expression of pro- and anti-apoptotic BCL-2 family members was determined.
**(A)** Western blot of BCL-2, BCL-X_L_ and BCL-X_S_, BID, BAD, BAX and BOK from lysates of ANDV-infected, HTNV-infected and uninfected cells after exposure to ABT-737. ANDV and HTNV nucleocapsid protein (ANDV-N; HTNV-N) were visualized using the monoclonal antibody 1C12. Calnexin was used as loading control. One representative experiment out of three independent experiments is shown.
**(B)** Fold change in total cellular BCL-2 and BCL-X_L_ in ANDV- and HTNV-infected cells compared to uninfected cells after treatment with ABT-737. Band densitometry analysis and the software ImageJ were used to measure the expression of the proteins and calculated as fold change increase compared to total cellular BCL-2 and BCL-X_L_ in uninfected cells at 72 hours p.i. Calnexin was used as loading control. Data shown represent the mean ± SD from three independent experiments.
**(C)** Fold change in total cellular BID, BAD, BAX and BOK in ANDV- and HTNV-infected cells compared to uninfected cells after exposure to ABT-737. Expression was measured by band densitometry analysis using the software ImageJ and calculated as fold change increase compared to total cellular BID, BAD, BAX and BOK in uninfected cells at 72 hours p.i. Calnexin was used as loading control. Data shown represent the mean ± SD from three independent experiments.
Paired t test was used for statistical evaluation: * p<0.05.
(TIF)

S4 Fig Neither treatment with ABT-737 nor BCL-2 knock down by siRNA cause significant changes in viral replication. A549 cells were infected with ANDV or HTNV at MOI 1. Cells were either treated with ABT-737 at approximately 56 hours p.i. and supernatants collected at 72 hours p.i., or transfected with siRNA targeting BCL-2 at 24 hours p.i. and supernatants collected at 48 and 72 hours p.i. Levels of progeny virus in supernatants were determined by titration.
**(A)** Titers of progeny virus in supernatants of ANDV-infected cells at 72 hours p.i., after treatment with ABT-737. Data shown represent the mean ± SD from three independent experiments.
**(B)** Progeny virus in supernatants from ANDV-infected cells after knock down of BCL-2 with siRNA. Titers were measured at 48 and 72 hours p.i. Data shown represent the mean ± SD from three independent experiments.
**(C)** HTNV progeny virus titers in supernatant of infected cells at 72 hours p.i. after treatment with ABT-737. Data shown represent the mean ± SD from three independent experiments.
**(D)** Progeny virus in supernatants from HTNV-infected cells after knock down of BCL-2 with siRNA. Titers were measured at 48 and 72 hours p.i. Data shown represent the mean ± SD from three independent experiments.
(TIF)
