# Supplementary figures and images for: Hantavirus inhibits apoptosis by preventing mitochondrial membrane potential loss through up-regulation of the pro-survival factor BCL-2
Source: PLoS Pathog. 2020 Feb 7;16(2):e1008297. doi: 10.1371/journal.ppat.1008297 (PMC7032725; doi:10.1371/journal.ppat.1008297)

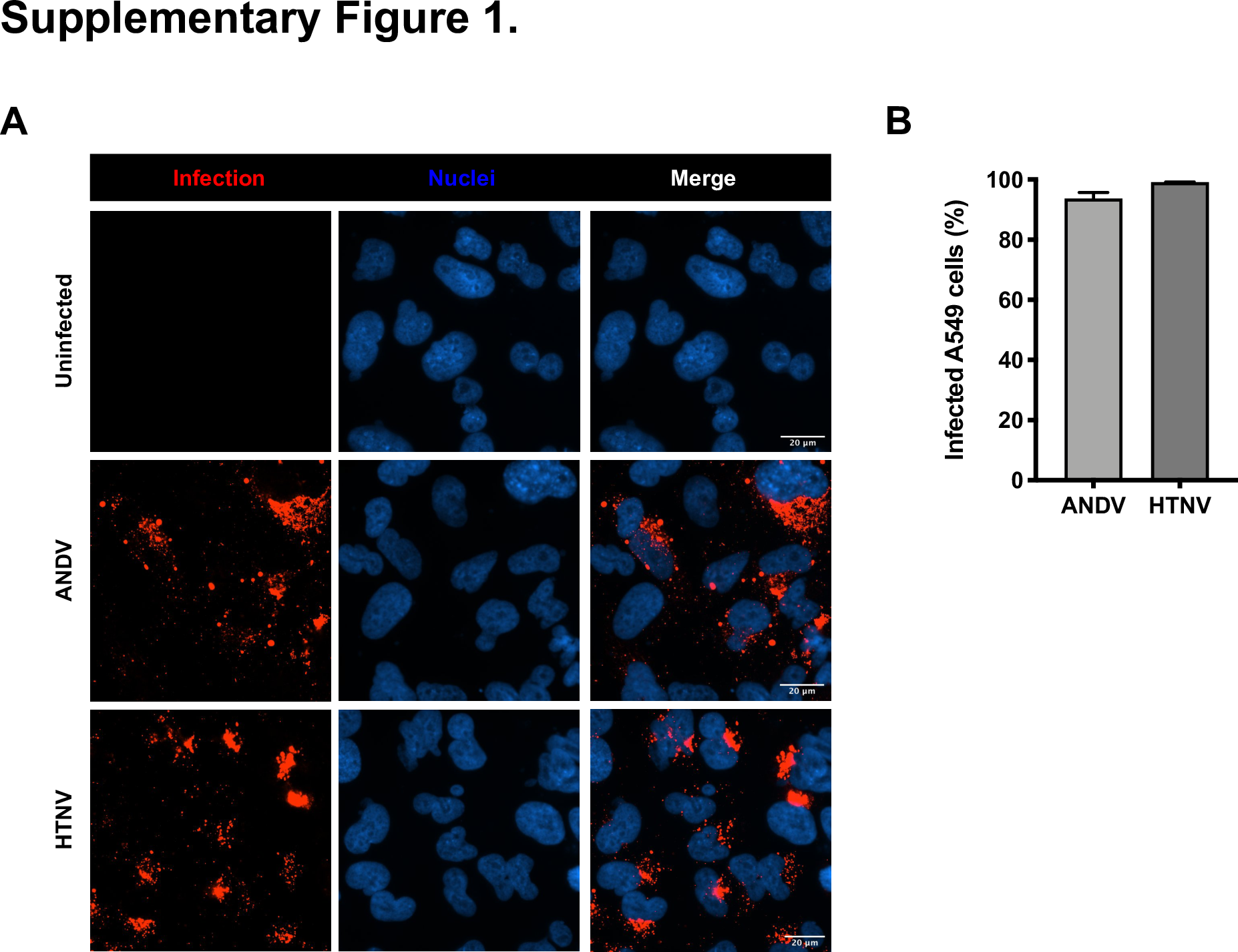

Supplement: S1 Fig — A549 cells were infected at MOI 1 for 72 hours. (A) Immunofluorescence images of ANDV-infected, HTNV-infected and uninfected cells. One representative experiment out of three independent experiments is shown. Scale bar, 20 μm. (B) Percentages of infected cells at 72 hours p.i. Numbers of infected and uninfected cells on the same slides were determined using fluorescence microscopy. Data shown represent the mean ± SD from three independent experiments in which at least 300 cells were counted for each experiment. (TIF) [file ppat.1008297.s001.tif]

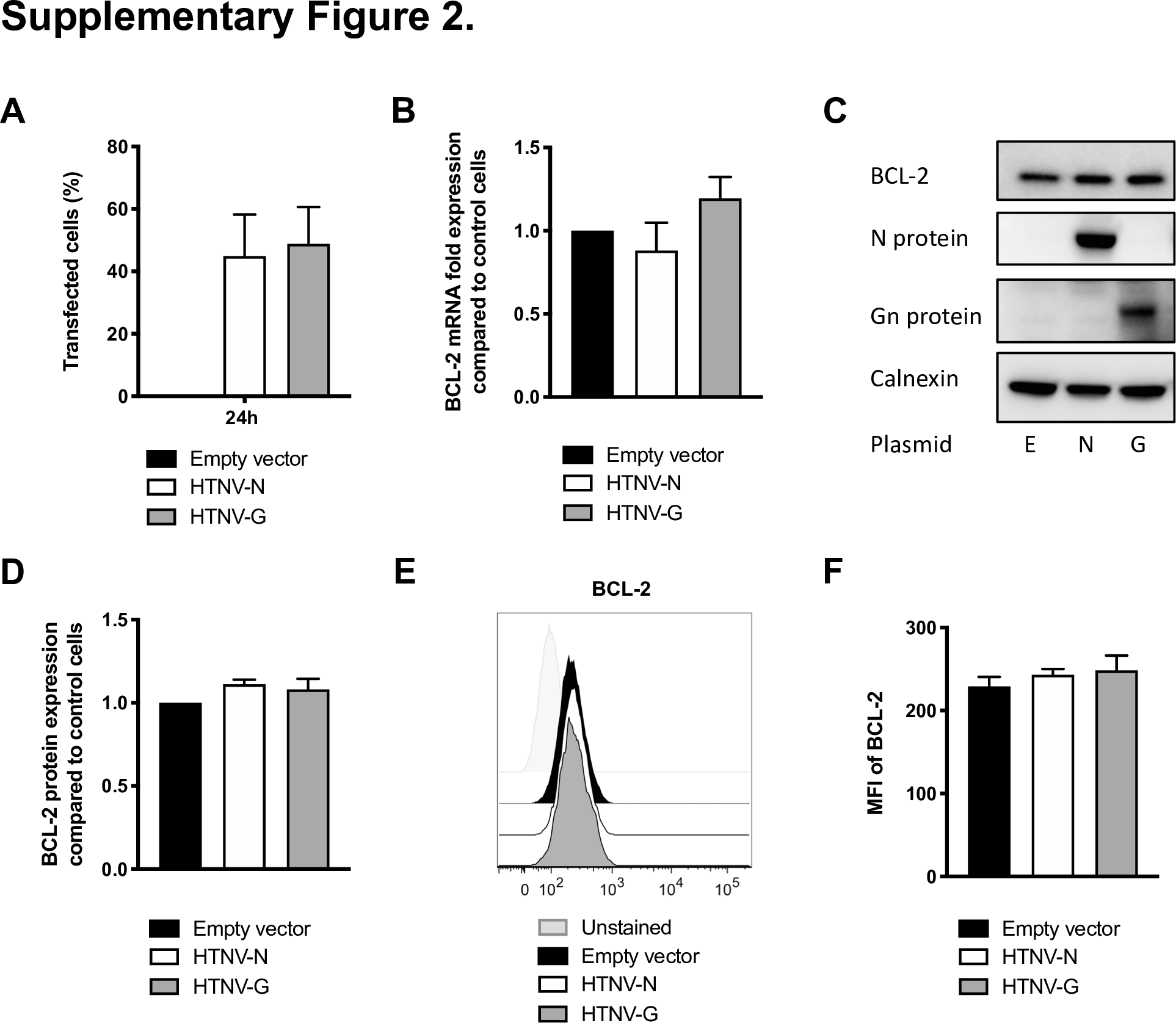

Supplement: S2 Fig — A549 cells were transfected with a plasmid expressing N protein, G proteins, or an empty vector as a control. (A) Transfection rates of plasmids expressing N and G proteins. Cells were transfected and 24 hours later stained with antibodies specific for N (mAb 1C12) or Gn (mAb 10B8) proteins. Numbers of transfected cells were determined using fluorescence microscopy, and percentage of N expressing and Gn expressing cells calculated. Data shown represent the mean ± SD from three independent experiments in which at least 300 cells were counted for each experiment. (B) mRNA expression levels of BCL-2 in N protein- and G protein-transfected cells compared to expression in empty plasmid-transfected cells. One representative experiment out of three independent experiments is shown. (C) Representative western blot of BCL-2 expression from lysates of transfected cells stained with antibodies specific for N (mAb 1C12) or Gn (mAb 10B8) proteins. Calnexin was used as loading control. One representative experiment out of three independent experiments is shown. (D) Fold change in total cellular BCL-2 in N or G expressing cells compared to cells transfected with an empty vector. Expression was measured by band densitometry analysis using the software ImageJ and calculated as fold change increase compared to total cellular BCL-2 in empty control cells. Calnexin was used as loading control. Data shown represent the mean ± SD from three independent experiments. (E) Representative histogram of flow cytometry analysis of BCL-2 expression in transfected cells after gating on N-expressing or G-expressing cells. One representative experiment out of three independent experiments is shown. (F) Flow cytometry analysis of BCL-2 shown as mean fluorescence intensity (MFI) in N-expressing or G-expressing cells compared to empty vector-transfected cells. Data shown represent the mean ± SD from three independent experiments. (TIF) [file ppat.1008297.s002.tif]

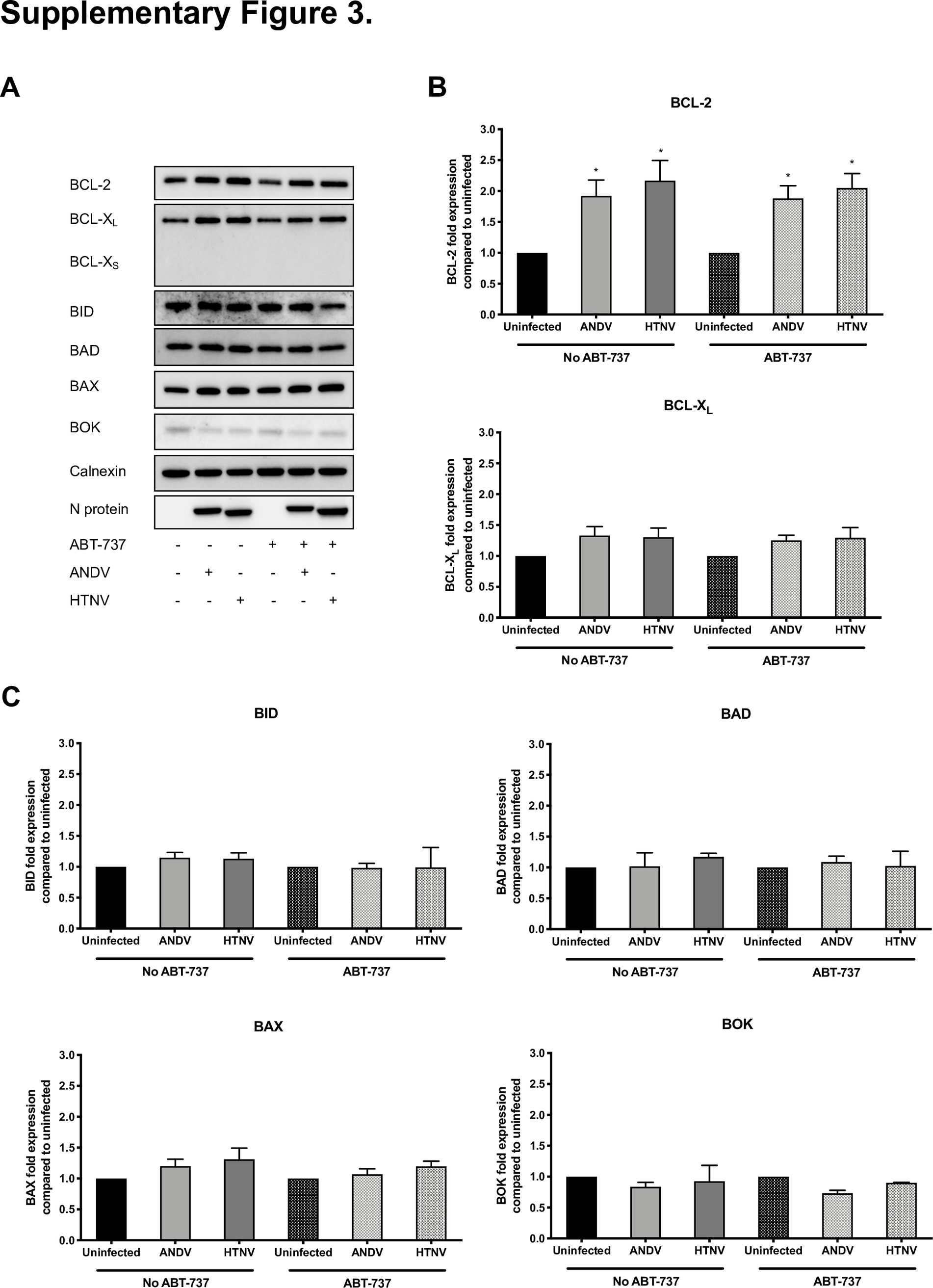

Supplement: S3 Fig — A549 cells were infected with ANDV or HTNV at MOI 1. Approximately at 56 hours p.i., the cells were treated with ABT-737 for 16 hours. At 72 hours p.i. the expression of pro- and anti-apoptotic BCL-2 family members was determined. (A) Western blot of BCL-2, BCL-XL and BCL-XS, BID, BAD, BAX and BOK from lysates of ANDV-infected, HTNV-infected and uninfected cells after exposure to ABT-737. ANDV and HTNV nucleocapsid protein (ANDV-N; HTNV-N) were visualized using the monoclonal antibody 1C12. Calnexin was used as loading control. One representative experiment out of three independent experiments is shown. (B) Fold change in total cellular BCL-2 and BCL-XL in ANDV- and HTNV-infected cells compared to uninfected cells after treatment with ABT-737. Band densitometry analysis and the software ImageJ were used to measure the expression of the proteins and calculated as fold change increase compared to total cellular BCL-2 and BCL-XL in uninfected cells at 72 hours p.i. Calnexin was used as loading control. Data shown represent the mean ± SD from three independent experiments. (C) Fold change in total cellular BID, BAD, BAX and BOK in ANDV- and HTNV-infected cells compared to uninfected cells after exposure to ABT-737. Expression was measured by band densitometry analysis using the software ImageJ and calculated as fold change increase compared to total cellular BID, BAD, BAX and BOK in uninfected cells at 72 hours p.i. Calnexin was used as loading control. Data shown represent the mean ± SD from three independent experiments. Paired t test was used for statistical evaluation: * p<0.05. (TIF) [file ppat.1008297.s003.tif]

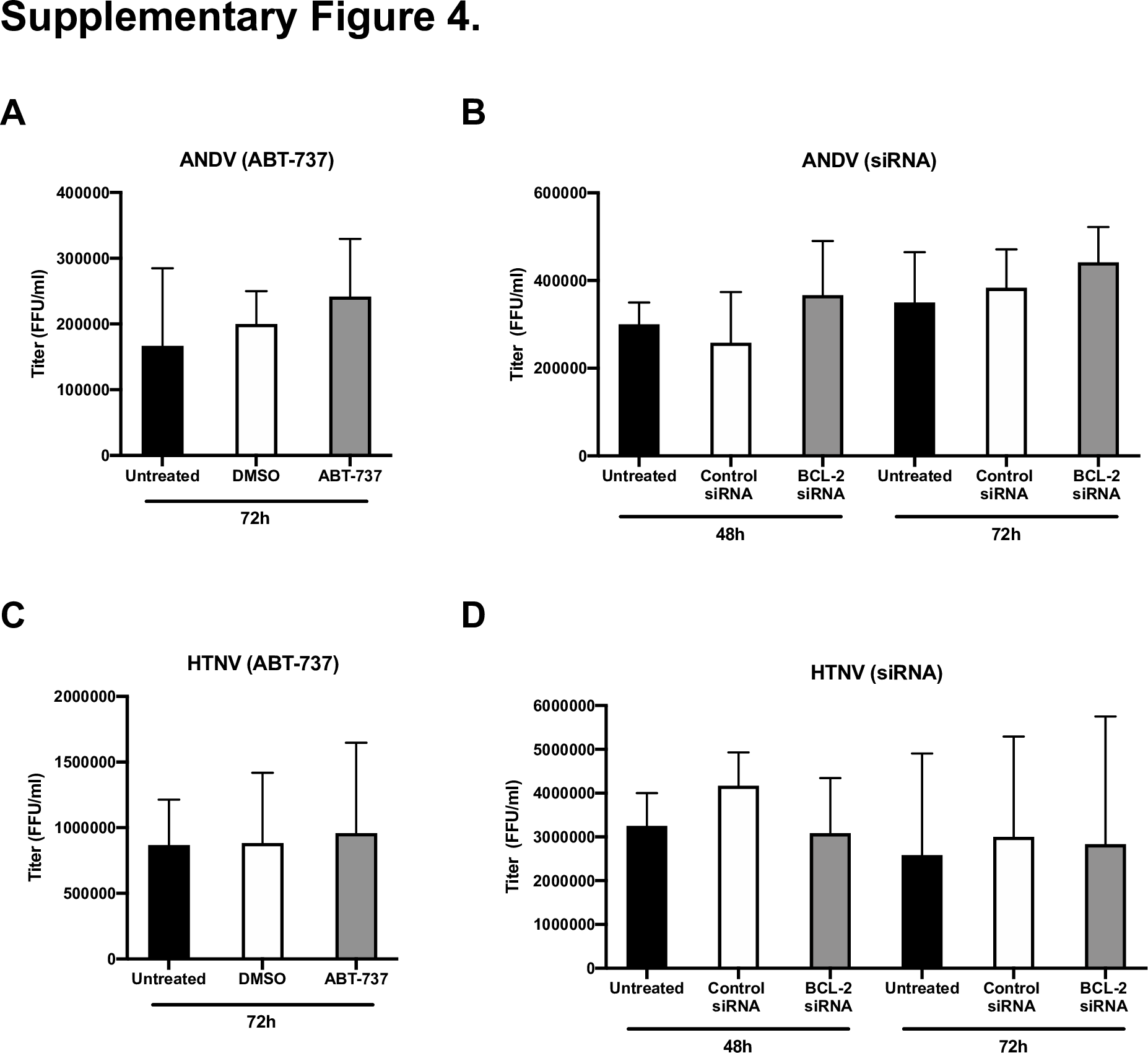

Supplement: S4 Fig — A549 cells were infected with ANDV or HTNV at MOI 1. Cells were either treated with ABT-737 at approximately 56 hours p.i. and supernatants collected at 72 hours p.i., or transfected with siRNA targeting BCL-2 at 24 hours p.i. and supernatants collected at 48 and 72 hours p.i. Levels of progeny virus in supernatants were determined by titration. (A) Titers of progeny virus in supernatants of ANDV-infected cells at 72 hours p.i., after treatment with ABT-737. Data shown represent the mean ± SD from three independent experiments. (B) Progeny virus in supernatants from ANDV-infected cells after knock down of BCL-2 with siRNA. Titers were measured at 48 and 72 hours p.i. Data shown represent the mean ± SD from three independent experiments. (C) HTNV progeny virus titers in supernatant of infected cells at 72 hours p.i. after treatment with ABT-737. Data shown represent the mean ± SD from three independent experiments. (D) Progeny virus in supernatants from HTNV-infected cells after knock down of BCL-2 with siRNA. Titers were measured at 48 and 72 hours p.i. Data shown represent the mean ± SD from three independent experiments. (TIF) [file ppat.1008297.s004.tif]
